# Supplementary material for: Development and Characterization of Symbiotic Buffalo Petit Suisse Cheese Utilizing Whey Retention and Inulin Incorporation
Source: Foods. 2023 Dec 1;12(23):4343. doi: 10.3390/foods12234343 (PMC10705983; doi:10.3390/foods12234343)
Supplement: Supplementary file 1 [file foods-12-04343-s001.zip › foods-2742608-supplementary.pdf]

foods MDPI

Table S1 presents the milk composition of Murrah buffalo milk

Table S1. - Murrah buffalo milk composition.

Parameter	Murrah buffalo milk
Fat (%)	5.32 ± 0.017
Protein (%)	3.51 ± 0.00
Lactose (%)	5.28 ± 0.005
Mineral Salts (%)	0.68 ± 0.00
Non-fat solids (%)	9.73 ± 0.005
Density (g/mL)	1.0333 ± 0.02
Electrical Conductivity (mS/cm)	4.43 ± 0.00
pH	6.68 ± 0.011
Acidity (% lactic acid)	0.16 ± 0.005
Added Water (%)	0

The given composition data for Murrah buffalo milk reflects that it has a fat content of 5.32%, which is indicative of the rich creaminess typical of buffalo milk, making it highly suitable for the production of high-fat dairy products like cheese and butter. The protein level at 3.51% suggests a high casein concentration, beneficial for cheese making and providing essential amino acids for nutrition. Lactose, at 5.28%, falls within the expected range, contributing to the milk's sweetness and calorie content, and is also a key factor in the fermentation process for dairy products. The mineral salts percentage of 0.68% indicates a healthy mineral content, including calcium and phosphorus, which are essential for the nutritional value of the milk. The non-fat solids, with a value of 9.73%, encompass lactose, proteins, and minerals, which are crucial in assessing the overall quality and processing suitability of the milk. A density of 1.0333 g/mL is typical for buffalo milk, reflecting its solid content and serving as a quick check against possible adulteration. The electrical conductivity at 4.43 mS/cm is a quality indicator, revealing the presence of various ions and can also serve as an indirect measure of udder health, particularly mastitis. The milk's pH stands at 6.68, slightly more acidic than cow's milk, which has implications for microbial growth, storage, and the processing of dairy products. An acidity level of 0.16% as lactic acid is within the normal range for fresh milk, influencing taste and shelf-life, and increasing with age or improper storage. Finally, the absence of added water confirms the milk's purity and unadulterated state, ensuring that the volume has not been artificially increased, thus maintaining its quality and integrity for consumption and processing.
